# The ‘communicatome’ of pregnancy: spotlight on cellular and extravesicular chimerism

**DOI:** 10.1038/s44321-024-00045-x

**Published:** 2024-03-11

**Authors:** Isabel Graf, Christopher Urbschat, Petra C Arck

**Affiliations:** 1 Division of Experimental Feto-Maternal Medicine, Department of Obstetrics and Fetal Medicine, University Medical Center Hamburg-Eppendorf, Hamburg, Germany; 2 Hamburg Center for Translational Immunology, University Medical Center Hamburg-Eppendorf, Hamburg, Germany

**Keywords:** Microchimerism, Pregnancy, Extracellular Vesicles, Feto-maternal Communication, Development, Urogenital System

## Abstract

Communication via biological mediators between mother and fetus are key to reproductive success and offspring’s future health. The repertoire of mediators coding signals between mother and fetus is broad and includes soluble factors, membrane-bound particles and immune as well as non-immune cells. Based on the emergence of technological advancements over the last years, considerable progress has been made toward deciphering the “communicatome” between fetus and mother during pregnancy and even after birth. In this context, pregnancy-associated chimerism has sparked the attention among immunologists, since chimeric cells—although low in number—are maintained in the allogeneic host (mother or fetus) for years after birth. Other non-cellular structures of chimerism, e.g. extracellular vesicles (EVs), are increasingly recognized as modulators of pregnancy outcome and offspring’s health. We here discuss the origin, distribution and function of pregnancy-acquired microchimerism and chimeric EVs in mother and offspring. We also highlight the pioneering concept of maternal microchimeric cell-derived EVs in offspring. Such insights expand the understanding of pregnancy-associated health or disease risks in mother and offspring.

## Abstract (web summary)

This Review discusses the origin, distribution, and function of pregnancy-acquired cellular chimerism and chimeric extracellular vesicles in mother and offspring.

## Graphical abstract

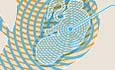

## Introduction

**Figure 1:**
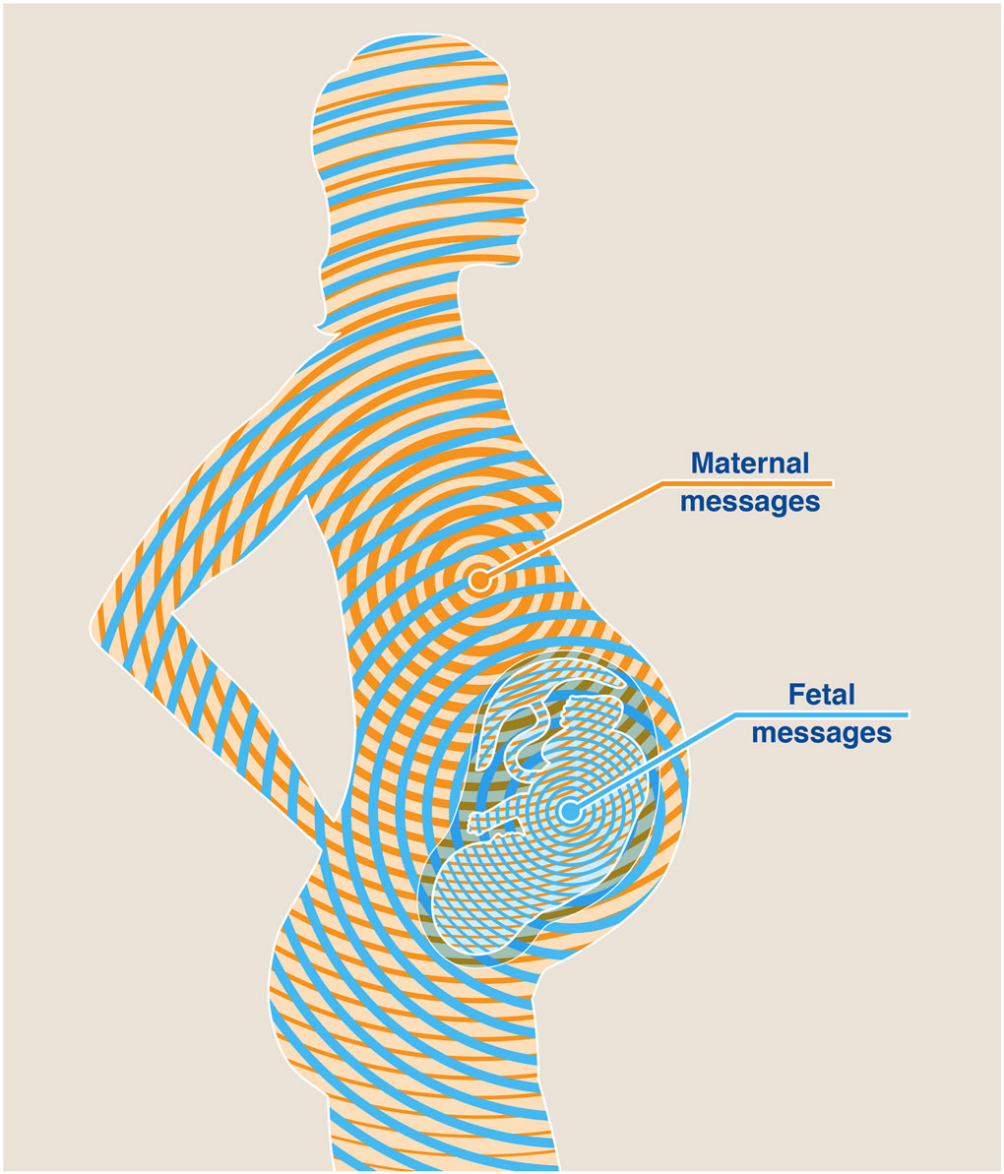
The “communicatome” of pregnancy. Maternal (marked in orange) as well as fetal (blue) mediators, e.g., antibodies, hormones, cells, extracellular vesicles, are vertically transferred during pregnancy. Due to the longevity of some of these mediators, e.g., cells, this feto-maternal communicatome can have long-lasting impacts on maternal and offspring’s health.

During pregnancy, fetal and maternal health is largely determined by the interaction—or communication—via biological mediators. These mediators are either secreted by the mother or the offspring and can be transferred from mother to fetus or vice versa (Fig. 1). Mediators involved in this feto-maternal communication network are multifaceted in their biological structure and longevity. Decades ago, it has been hypothesized that such mediators also include “substances of immunologic importance”, such as antibodies which are being transferred from mother to fetus (Page, 1957). These could subsequently be specified as pathogen-specific antibodies, which are of “immunological importance” by mitigating the risk for early life infections of neonates (Hay et al, 1971; Madani and Heiner, 1989; Albrecht et al, 2022).

In addition to transiently available markers such as maternal antibodies—which wane 4–6 months after birth—a new layer of complexity within the feto-maternal “communicatome” arose from the discovery of pregnancy-associated chimerism (Owen, 1945; Billingham et al, 1953). Chimerism is defined as the presence of genetically distinct cells or DNA originating from another individual. During pregnancy, chimerism occurs naturally, opposed to e.g., chimerism induced by blood transfusion or transplantation (Mathe et al, 1963). The currently available detection methods of chimeric cells in mother and fetus indicate a frequency of less than one percent of all cells (Hall et al, 1995; Nelson, 2012; Stelzer et al, 2021). Hence, the term microchimeric cells (MC) was coined. Intriguingly, not only cells but also extracellular vesicles (EVs) can be vertically transferred between mother and fetus and add to pregnancy-associated chimerism. EVs are lipid-membrane-bound particles in the size range of nano- to micrometer and continuously secreted by a wealth of cell types into the extracellular space (Smith et al, 1974; Knight et al, 1998; Théry et al, 2018; Sheller-Miller et al, 2019).

**Figure 2:**
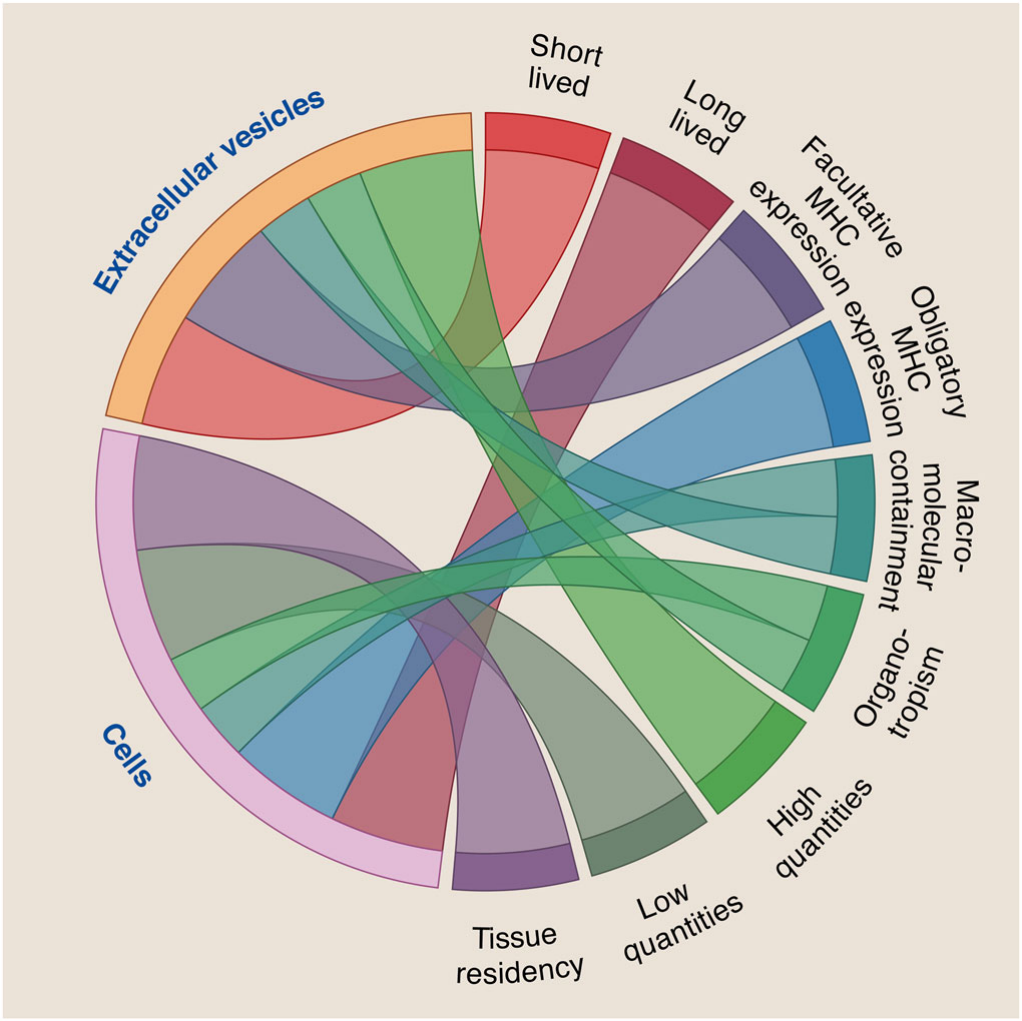
The chimeric coding systems—features and functions. Characteristics of the pregnancy-acquired chimeric coding systems, focusing on extracellular vesicles and cells (left) and their associated characteristics, features and functions (right).

Thus, not only pregnancy-derived microchimeric cells but also EVs are increasingly recognized as key messengers within the pregnancy-associated communicatome. Hereby, the EVs’ and MCs’ genetic demarcation from the host—even if this host is closely related—has sparked the interest of immunologists from various fields. However, their cargo, which can include proteins, lipids, RNA and also DNA, has not been fully decoded. This currently limits our understanding of their impact as part of the multilayered ‘coding systems’ within the communicatome—the coding of biological messages. Key features of pregnancy-derived microchimeric cells and EVs include the carrying of major histocompatibility complex (MHC) molecules or their ability to target specific organs (Fig. 2) (Nguyen et al, 2021; Stelzer et al, 2021; Schepanski et al, 2022; Buzas, 2023). Microchimeric cells have the exclusive feature of considerable longevity and can be detected in the host up to decades (Bianchi et al, 1996; Maloney et al, 1999). We here critically review the pregnancy-associated chimeric coding systems and highlight their key functions.

### Cellular microchimerism—pregnancy’s gift?

Cellular chimerism can be induced by medical interventions, such as transplantation of solid organs or stem cells from a donor into an allogenic recipient. The only form of naturally acquired chimerism occurs in the context of pregnancy from the bidirectional transfer of cells between mother and fetus. Besides the cellular exchange between mother and fetus, it is long known that chimerism also occurs between twins, often resulting from vascular anastomoses. This was first described in bovine pregnancies and created the foundation of modern chimerism research (Owen, 1945). Based on these findings several studies investigated the occurrence of in utero chimerism between twins in humans. Interestingly, one case report describes cells expressing non-inherited male antigens in an adult male. Since this man had no siblings, the most likely source for those cells may be an unrecognized “vanished” twin (de bellefon et al, 2010).

The presence of fetal cells in the maternal organism is referred to as fetal microchimerism. Vice versa, the presence of maternal cells in the fetus is termed maternal microchimerism. Both, fetal microchimeric cells (FMc) and maternal microchimeric cells (MMc) can not only be detected during pregnancy, but also long after birth in the respective host (Maloney et al, 1999; O’Donoghue, 2008). Intriguingly, pregnancy-acquired microchimerism can create a pool of genetically foreign cells within an organism. For example, the pool of FMc increases with each subsequent pregnancy in the maternal body, hereby expanding the diversity of FMc in the mother. In consequence, younger siblings are considered to receive a more diverse composition of MMc, which not only includes maternal cells, but can also contain cells from older siblings, or their grandmother and possibly even elder siblings of their mother (uncles, aunts) (Kinder et al, 2017; Shao et al, 2023). This pool of trans- and intergenerational microchimeric cells within an organism has been termed microchiome.

Pregnancy-associated microchimerism is not limited to the human species but also present in a large number of placental animals. However, the presence of pregnancy-associated chimerism in non-placental mammalian species, e.g., monotremes like platypus, or in species with very short-lived placentae such as marsupials has not been assessed to date. Given that these species also breastfeed their offspring, breast milk-derived maternal chimerism can be assumed, but its function is unknown. Nevertheless, the presence of microchimeric cells in different eutherian placental mammals underpins that pregnancy-induced microchimerism is a highly conserved phenomenon which has not been eliminated during evolution (Boddy et al, 2015). Intriguingly, placental diversity between species (hemochorial, epithelial, endothelial) plays a minor role, as microchimerism has also been found in primates, dogs, cows and mice (Bryan, 2015; Lindtke et al, 2023). Since human studies have known limitations, especially when it comes to obtaining biological samples in a pregnancy cohort, mice represent an excellent model organism for studying pregnancy-related microchimerism. In addition to the high anatomical similarity between human and murine placentas (Hemberger et al, 2020) humans and mice share also around 90% (Soncin et al, 2018) of the genes leading to comparable physiological and immune responses.

In human and mouse, MMc and FMc comprise of a broad range of cells, including progenitor cells, fully differentiated immune cells, stroma cells and stem cells (Kinder et al, 2017; Fujimoto et al, 2022). Although being genetically distinct, microchimeric cells are not being rejected. One of the seminal studies in the field of maternal microchimerism highlighted that the human fetal T-cell response against non-inherited maternal antigens (NIMA) expressed on MMc is suppressed by fetal regulatory T-cell (T_reg_)-dependent pathways (Mold et al, 2008). This can explain why NIMA^+^ maternal cells are not rejected by the fetal immune system. Similar pathways have been identified in mice, as the percentage of organs harboring MMc correlates with both, lymphoproliferation and host T_reg_ activity. However, it is puzzling that T_reg_-mediated tolerance is also detectable against paternal alloantigens, as shown in adolescent humans (Mold et al, 2008). Evidence from preclinical models suggests that previous pregnancies promote the generation of immune tolerance for subsequent pregnancies (Kinder et al, 2015). This “advanced” immune tolerance is mediated by the expansion of NIMA-specific T_reg_ cells in the female fetus in mice, accompanied by the long-term plasticity of FoxP3 expression. Interestingly, in mice MMc-associated expansion of NIMA-specific T_reg_ cells are being eliminated once these female offspring become pregnant themselves, suggesting a reprogramming of pregnancy-imprinted immunological memory (Thiele et al, 2019; Shao et al, 2023).

### Communication pathways and functional roles of microchimeric cells

#### Spotlight on maternal microchimerism

Published insights underpin that fetal and maternal microchimerism fundamentally differ in their function. These functions seem to depend on the tissue environment in which they are engrafted. The cellular phenotype of MMc in human and mice is a broad and includes T, B, NK cell subsets, monocytes/macrophages, granulocytes and stem cells (Loubière et al, 2006; Cuddapah Sunku et al, 2010; Kinder et al, 2017). Intriguingly, the latter seem to be capable of differentiating into organ-specific cells, as echoed by the presence of female cardiac cells (likely of maternal origin) in human male infants who succumbed to heart block (Stevens et al, 2003). Besides heart tissue, the presence of MMc could also be confirmed in offspring’s bone marrow, thymus, lungs, heart, pancreas, liver, lymph nodes, spleen, kidney, adrenal gland, ovary, testes, and brain in various mammalian species (Jonsson et al, 2008; Stelzer et al, 2021; Schepanski et al, 2022).

MMc have been often studied in diseased individuals, e.g., patients with various autoimmune disorders. One example is the detection of female insulin-producing beta cells in the pancreas of males suffering from diabetes type I (Vanzyl et al, 2010). Such studies have nourished the notion that MMc are capable of replacing damaged or eliminated cells. However, there is still ambiguity about this interpretation, since evidence for the presence of maternal cells was also found in pancreatic tissue of healthy males (Nelson et al, 2007). This also supports the interpretation that maternal cells in offspring’s tissue may in fact trigger autoreactive responses. This interpretation is supported by observations in patients with biliary atresia (Muraji et al, 2022), a disease prevalent in neonates where the bile ducts are blocked. These neonates show significantly elevated levels of bilirubin already at birth (Harpavat et al, 2011), underlining an onset of biliary atresia before birth. In the liver of affected offspring, MMc have been identified as biliary epithelial cells, but also as effector T cells. This led to the suggestion that the presence of MMc in the fetal bile duct causes cholangitis, which then leads to liver failure (Leveque et al, 2014; Muraji et al, 2022).

**Figure 3:**
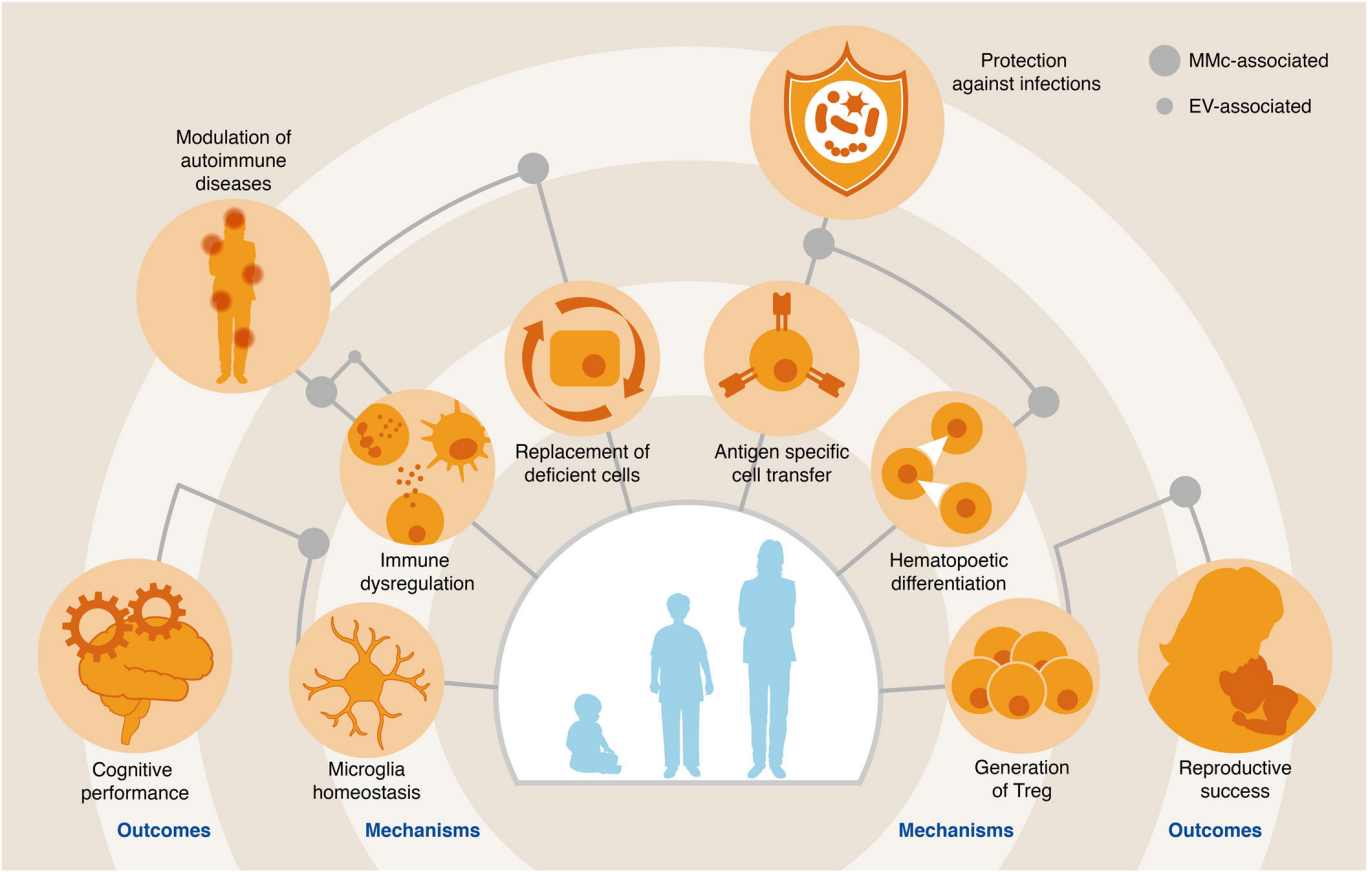
Implications of maternal microchimeric cells and chimeric extracellular vesicles. Pregnancy-associated (micro-)chimerism is associated with numerous consequences for fetal health. The inner circle depicts mechanisms by which corresponding outcomes—displayed in the outer circle—are promoted by either EVs (small dots), microchimeric cells (large dots), or both. For example, there is a MMc-associated modulation of hematopoetic differentiation, which protects the infant against infections.

Recent findings arising from our group provided insights into MMc-mediated protection of human offspring from severe respiratory infections. Respiratory infections during the first year of life were found to be inversely correlated with the number of MMc in the umbilical cord blood at birth, especially in male children. Remarkably, this MMc-associated protection occurred during the second half of the first year of life, when passive immunity mediated by the transplacental transfer of pathogen-specific maternal antibodies has waned. The reduced risk of infection in human offspring attributed to MMc could be seconded by functional experiments in mouse models and provided insights into the mechanisms underlying this protection. Here, experimental modulation of MMc numbers in murine offspring could confirm that MMc promote the differentiation of hematopoietic stem cells into myeloid cells, which can subsequently bolster the innate immune response upon pathogen encounter (Stelzer et al, 2021). We were also able to identify that MMc can mitigate the risk of severe infection in offspring via antigen-specific pathways, as we could confirm the transplacental transfer of maternal pathogen-specific CD8^+^ T cells in mice (Yüzen et al, 2023). The risk for childhood malaria has also been studied in the context of MMc, which unearthed that children who contracted malaria via the placenta in cases of maternal malaria infection had a higher risk to get infected during early childhood, whilst the course of the disease was milder compared to children affected by malaria without prenatal exposure (Harrington et al, 2017). Children exposed to prenatal malaria had a higher number of MMc, suggesting that MMc provide protection against severe malaria. Yet, it remains elusive to what extent maternal antibodies contribute to this protection. Another example supporting a protective function of MMc for offspring’s health is the MMc-dependent promotion of brain development and cognitive abilities. In murine preclinical models, MMc suppressed microglia activation and reduced the elimination of presynaptic vesicles, which was linked to improved behavioral abilities (Schepanski et al, 2022). The wide range of MMc-associated protective and harmful implications on fetal health are summarized in Fig. 3.

#### Spotlight on fetal microchimerism

**Figure 4:**
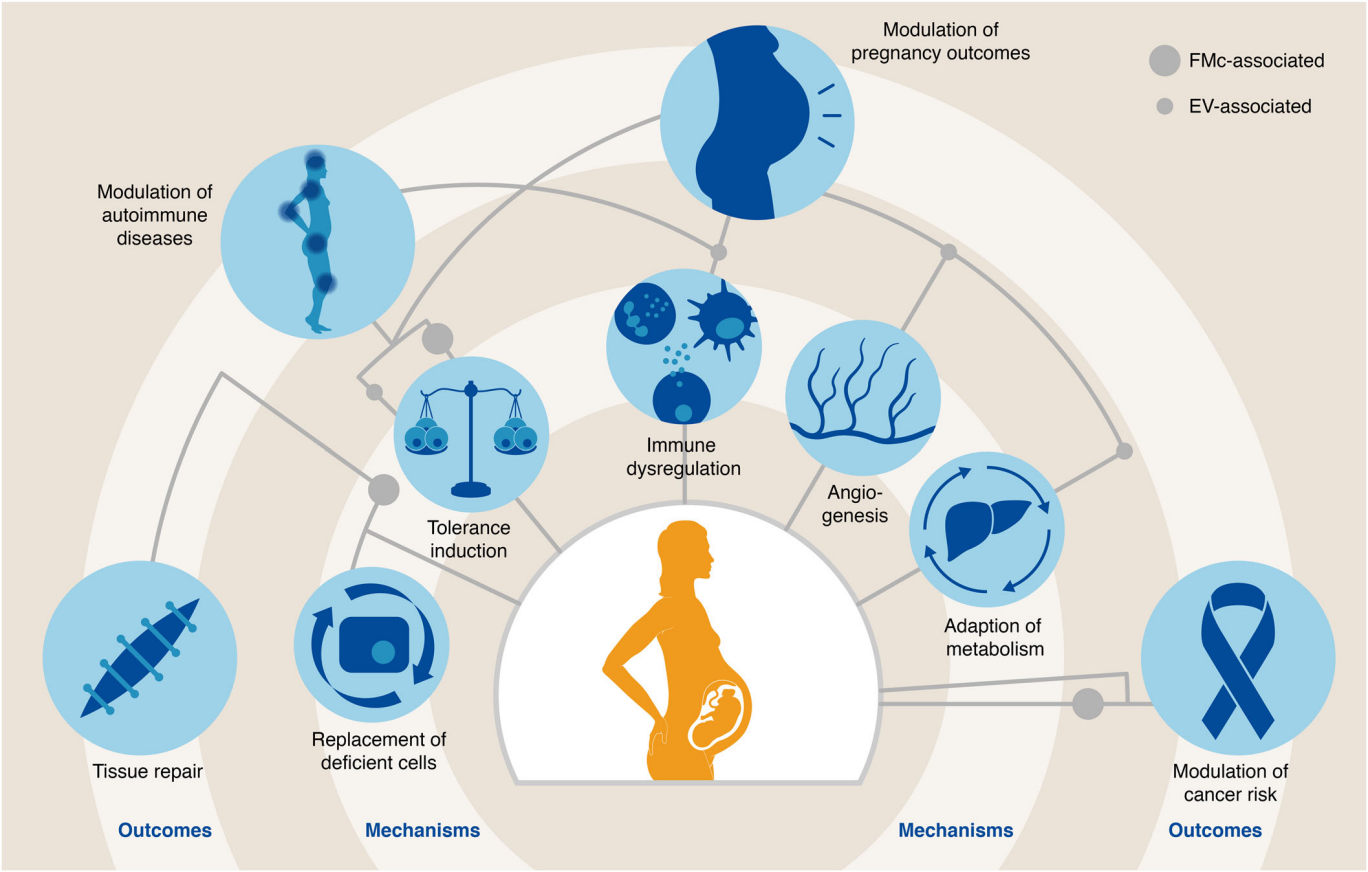
Implications of fetal microchimeric cells and chimeric extracellular vesicles. Pregnancy-associated (micro-)chimerism is associated with numerous consequences for maternal health. The inner circle depicts mechanisms by which corresponding outcomes—displayed in the outer circle—are promoted by either EVs (small dots), microchimeric cells (large dots), or both. For example, an EV-associated immune dysregulation is connected to the modulation of pregnancy outcomes as well as autoimmune diseases.

FMc have been first described more than a century ago in lung tissue of women with pre-eclampsia (Schmorl, 1893). In uncomplicated pregnancies, FMc begin to appear in maternal blood as early as four to five weeks of gestation and steadily increase throughout gestation, reaching a maximum at birth (Thomas et al, 1994; Lo et al, 1998; Ariga et al, 2001). FMc engraft in a number of maternal organs and tissue structures. Due to the relative immaturity of the fetal immune system, the FMc pool is likely less heterogenous compared to the pool of MMc. Similar to MMc, the population of human FMc also comprises of various cell types, including T cells, B cells, monocytes/macrophages, NK cells, granulocytes, but also hematopoietic and mesenchymal stem cells (Loubière et al, 2006; Kinder et al, 2017). Hematopoietic and mesenchymal stem cells contribute to the majority of the FMc pool during pregnancy in the maternal bloodstream (Bianchi, 1999; Osada et al, 2001). Hence, fully differentiated fetal cells that can be detected in maternal tissues years or even decades after pregnancy may have differentiated from fetal-derived stem cells (Johnson et al, 2002; O’Donoghue, 2008). However, their functional role is intensely debated and potential functions cover a broad repertoire of functions as depicted in Fig. 4, ranging from advantageous health effects such as tissues repair, to disadvantageous health consequences, e.g., autoimmune reactions and the progression of cancer (Lambert and Lee Nelson, 2003; Gilmore et al, 2008; Boddy et al, 2015; Sedov et al, 2022).

The latter is supported by the detection of FMc in various malignant tumors, e.g., in the breast, colon, brain, lung, thyroid, cervix and skin (Cirello et al, 2008; Dubernard et al, 2008; Broestl et al, 2018). This sparked research aiming to identify possible functional roles of FMc in modulating cancer risk. For example, the presence of FMc in maternal blood could be linked to a decreased risk for breast cancer, whilst FMc positively correlated with the risk for colon cancer (Kamper-Jørgensen et al, 2012). Intriguingly, increased parity—and hence, a greater number or heterogeneity of FMc—was linked to a lower risk for brain cancer (Chiu et al, 2012; Kamper-Jørgensen et al, 2012). However, other studies report a high correlation between FMc and glioblastoma (Broestl et al, 2018), suggesting that FMc may increase the risk for brain tumors. The presence of FMc has also been detected in human primary melanomas during pregnancy, whereas benign skin lesions like nevi were largely devoid of FMc (Nguyen Huu et al, 2009b). Strikingly, the majority of these FMc expressed endothelial progenitor markers, suggesting that FMc promote lymphangiogenesis, which may in turn also promote the progression of the maternal skin cancer. Thus, a wealth of published evidence on the potential functional role of FMc in modulating the risk for various malignancies is available, although often correlative, anecdotal, circumstantial, and, furthermore, studies all too often differ in the tissue examined. While some studies examined FMc levels in tissue samples taken from the cancer site, other studies used blood. To date, it is unclear to what extent FMc levels in the blood provide valid information about the role of FMc in the development of certain tumors. FMc could be recruited from the periphery to the site of carcinogenesis to fight cancer growth, which in turn leads to lower levels of FMc in the blood, which would support the errorous interpretation that low levels of FMc are linked to a higher cancer risk. Hence, a thorough analysis of the functional role of FMc with regard to distinct tumor entities, including the consideration of known confounders—for example, that the reduced risk for breast cancer may result from breast feeding rather than the presence of FMc—as well as standardization and harmonization of examination protocols in order to avoid methodological differences between studies are urgently needed.

Besides the risk for cancer, also the response to immunotherapies might be modulated by FMc. An essential factor that drives cancer development, disease outcome and immunotherapy success is the sexual dimorphism observed in various cancer types. It is known that males have a higher risk to develop cancer followed by a twofold higher mortality rate (Haupt et al, 2021). Studies that investigated the tumor microenvironment of early-stage non-small cell lung cancer have shown that the tumor microenvironment of females exhibited a higher degree of inflammation compared to males, suggesting a more effective tumor response (Conforti et al, 2021). However, when women undergo immunocheckpoint inhibition therapies, the outcome was worse compared to men. Since FMc are exclusively present in women, they should be considered as modulators in cancer types with a female predominance, excluding cancer of reproductive organs. For example, the incidence of melanoma is approximately twice as high in women than in men until the age of 50 (Noone et al, 2018), and is the most diagnosed malignancy in pregnant women in western countries (Wong et al, 1989; Lishner, 2003). Paralleling this, in mouse as well as human studies FMc have been found to invade melanomas (Nguyen Huu et al, 2009a). Clearly, other confounding factors of tumor development and progression, such as lifestyle factors and sex hormones, must also be taken into account (Straub, 2007; Tyagi et al, 2012).

In addition, tissue repair functions have also been attributed to FMc, such as the promotion of vascularization in injured maternal tissues. This has been described in mouse models of maternal skin fibrosis, focusing on fetal endothelial progenitor cells (Badiavas, 2004; Nassar et al, 2012). In preclinical murine models of cardiac injury, FMc have been shown to engraft the injured maternal heart and adopt diverse phenotypes, ranging from cardiac lineage endothelial cells, smooth muscle cells, and cardiomyocytes (Kara et al, 2012), which also supports their potential tissue repair functions.

In summary, the bidirectional exchange of cells between mother and fetus during pregnancy and subsequent pregnancy-associated microchimerism in mother or fetus has divergent, sometimes contradictory consequences for maternal and offspring’s health. As outlined above MMc and FMc are e.g., capable of replacing deficient cells in the respective host. However, since the frequency of MMc and FMc is very low, the extent of such replacement in mitigating the onset or course of disease is still unknown. Since maternal cells have been suggested to replace pancreatic islet beta cells in male offspring, the confirmation that the maternal cells are indeed capable of producing insulin and hereby reducing blood glucose levels in diabetic offspring would be an excellent example for MMc-mediated mitigation of offspring’s health. Given the rapid emergence of the field of microchimerism in publications and research networks, along with advanced technologies that allow to assess even small number of cells in depth, including their phenotype and function, we can expect that the gaps in knowledge related to the enigma of pregnancy-acquired microchimerism will be filled in the near future.

### EV-mediated communication from the feto-placental unit

#### Biogenesis and organotropism of placenta-derived EVs

As mentioned above, EVs can be vertically transferred between mother and fetus, hereby contributing to pregnancy-associated chimerism. EVs are spherical nano-scaled particles, enclosed by a lipid bilayer harboring biomolecules, and can be continuously secreted by almost every cell type into the extracellular space (Smith et al, 1974; Théry et al, 2018). Fetal tissues—such as the placenta—release EVs into the maternal circulation (Knight et al, 1998; Redman and Sargent, 2000; Sarker et al, 2014; Tong and Chamley, 2018). Here, EVs—together with FMc—contribute to the microchiome. Conversely, EVs derived from maternal tissues can also reach the fetus, similar to MMc (Sheller-Miller et al, 2019; Kaisanlahti et al, 2023).

**Figure 5:**
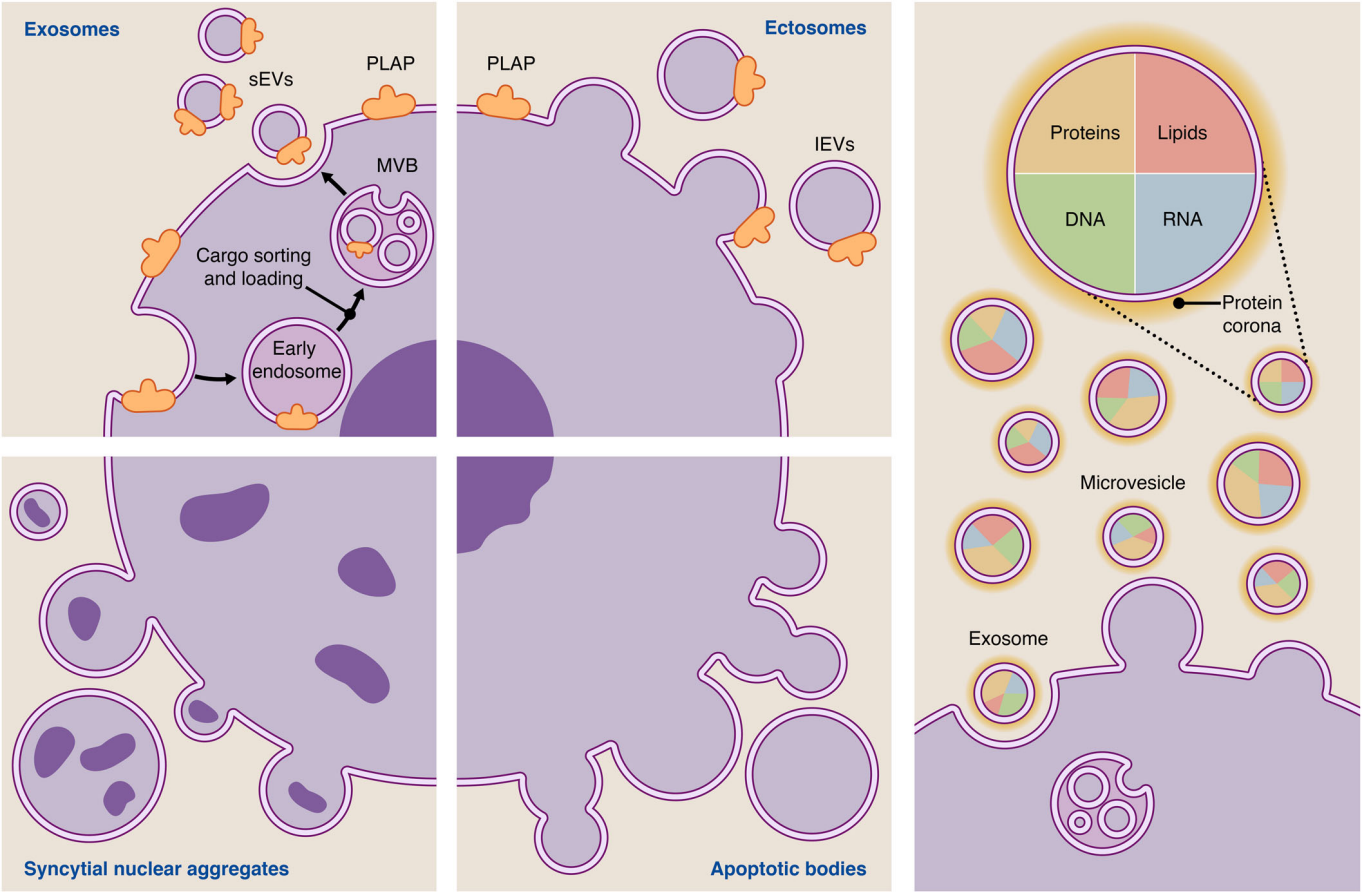
Extracellular vesicles—biogenesis and cargo. There are four major subtypes of vesicles released into the extracellular space when referring to placenta-associated EVs: exosomes, ectosomes, apoptotic bodies and syncytial nuclear aggregates (left part of this figure). Exosomes are generated via the endosomal pathway (left top). Starting point is the production of early endosomes by invagination of the cell plasma membrane containing plasma membrane proteins as well as extracellular proteins and eventually they mature to late endosomes (Mathieu et al, 2019). The endosomes are then loaded with proteins originating from the trans golgi network as well as the endoplasmatic reticulum and vice versa release proteins to these compartments. Next, by inward budding of the membrane of the late endosomes multi vesicular bodies (MVB) arise, which form the EV precursors. Fusion of MVB with the plasma membrane finally leads to release of EVs termed as exosomes, which are in the size range of around 50–150 nm. The second major EV biogenesis pathway consists of outward budding of the plasma membrane with consecutive shedding of the vesicle (right top). Also here cargo sorting mechanisms direct the future cargo of the EVs to the plasma membrane (Tricarico et al, 2017). These EVs are termed ectosomes with the microvesicles being their best studied representative and range from 200 nm up to >1000 nm. Among the exosomes and ectosomes a considerable number of subpopulations has been identified: large oncosomes, exophers, mirgasomes—just to mention a few (Buzas, 2023). The apoptosis of a cell leads to the third major EV population: apoptotic bodies (right buttom). They are rather variable in content and size and range from 100–5000 nm. Lastly, the outer layer of the placenta—the syncytiotrophoblast, which can be characterized by its multinuclear syncytium—secretes vesicles larger >20 µm containing several nuclei (left bottom) (Huppertz et al, 2002; Tong et al, 2018). The cargo of EVs includes proteins, lipids, DNA and RNA (right part of this figure). The cargo varies depending on the specific EV subtype. In addition, it has been suggested that EVs are surrounded by a protein corona referring to proteins and other macromolecules, which are externally attached to the EV and form a surrounding layer (Heidarzadeh et al, 2023). These macromolecules might spontaneously adsorb to the EVs’ surface and are acquired from protein-rich environments such as plasma (Tóth et al, 2021). PLAP placental alkaline phosphatase, sEVs small EVs, lEVs large EVs.

EVs is an umbrella term for a variety of subtypes, which differ in their characteristics, such as cellular origin and size. As depicted in Fig. 5, these subtypes differ with regard to their cargo and function (Huppertz et al, 2002; Choi et al, 2015; Ouyang et al, 2016; Tong et al, 2016). However, it is challenging to differentiate between such subtypes, e.g., exosomes and ectosomes. In order to increase comparability within studies, is has been proposed to term EVs based on their size into small EVs (sEVs originally referred to as exosomes) and large Evs (lEVs originally referred to as microvesicles), unless their origin can be clearly identified (Théry et al, 2018).

Upon release, the EVs can function in an autocrine, paracrine or endocrine manner (Crewe et al, 2018; Zhang et al, 2022; Kulaj et al, 2023). In the context of pregnancy, placental EVs are required to pass the extracellular matrix (ECM) and the endothelial cell barrier prior to reaching the blood vessels (Amenta et al, 1986; Lee et al, 1993; Kupper and Huppertz, 2022; Debnath et al, 2023). The exact mechanism how EVs navigate through the interstitium and overcome the endothelial barrier to enter the circulation—either the blood or the lymphatic circulation is still unknown (Debnath et al, 2023; Iannotta et al, 2024). However, once EVs have reached the bloodstream, EVs have been proposed to be present in plasma for approx. 40 min (Auber and Svenningsen, 2022) before potentially entering maternal organs (Nguyen et al, 2021; Kang et al, 2023).

Intriguingly, there is evidence that besides non-specific uptake of EVs, EVs can migrate into specific target organs. In a groundbreaking study, EVs derived from distinct tumor entities showed a preferential organotropism depending on the tumor entity in mouse (Hoshino et al, 2015). This EV organotropism correlated with specific integrin expression profiles, e.g., integrin alpha 6 (ITGα_6_) as well as ITGβ_4_ and ITGβ_1_ were highly abundant in lung-tropic EV. This specific integrin expression profiles differed from the expression profile of the originating tumor, which underpins that this parent tissue selective packaged the EVs. Preclinical models provide first evidence that the presence of integrins on placental EVs (subtype not specified) also promotes their migration to other tissues implying the possibility of specific targeting of placental EVs (Nguyen et al, 2021). This is underscored by emerging evidence supporting that placental EV seed into the maternal lung, liver, kidney and spleen (Tong et al, 2017a; Tong et al, 2017b; Tsai et al, 2022). However, the heterogeneous source of EVs used—e.g., EVs from plasma or placental explants, from mice or human—its concentration and the timepoint of assessment greatly differ between studies and will have to be controlled for in future studies (Kang et al, 2023).

This is also a challenging task when discriminating the origin of placental EV. The placenta is a large and complex organ, which functions as fetal lung (oxygen supply), liver (metabolism), kidney (elimination of waste products) and GI tract (nutrition) at the same time. Discrimination of placental EVs originating from which of various placental cellular subtypes is currently not possible due to the lack of knowledge on suitable biomarkers for EV subtypes (Arutyunyan et al, 2023). To date, placental alkaline phosphatase (PLAP), Syncytin-1 and human leukocyte antigen (HLA)-G, all membrane-bound proteins, function as marker of placental EVs. The most commonly used placental EV marker is PLAP, which is highly abundant in the syncytiotrophoblast and cytotrophoblast. However, PLAP is also expressed in fetal membranes, such as the amniotic epithelium and the chorionic trophoblast, as well as in decidual and myometrial cells, mesenchymal cells, and cervical glands (Leitner et al, 2001; Dixon et al, 2018; Basiri and Pahlavanneshan, 2021). This broad expression must be taken into consideration when using PLAP for the detection of placental EVs. Noteworthy, PLAP is also detectable in non-pregnant females and males, most likely due to an intestinal isoform (Li et al, 2023). Compared to PLAP, syncytin-1 has been used in fewer studies, but is also expressed in rodent placentas (Han et al, 2020). Lastly, HLA-G, an immunomodulatory molecule highly specific for the placenta, might not be the most suitable marker for placental EVs, since it decreases towards term and is not detectable in third trimester placental explants as well as was exclusively detected in small, but not large EVs by mass spectrometric analyses (Kshirsagar et al, 2012; Taylor et al, 2020).

#### Communication pathways and functional roles of placental EV

Once the placental EVs seed into maternal organs, several mechanisms are in place to communicate their message and convey their function. Hereby, EVs interact with cell surface molecules of the recipient cell in a receptor-ligand fashion, or are being internalized by the recipient cell. This internalization of EVs is an orchestrated process involving different uptake routes, including macro- and micropinocytosis, phagocytosis, and endocytosis (Mathieu et al, 2019). There is currently no consensus on the preferred uptake mechanism of specific EVs (Mathieu et al, 2019). This is specifically relevant during pregnancy, as the pathways of placental EV uptake in maternal organs are still underexplored (Cronqvist et al, 2020; Li et al, 2020; Feng et al, 2022). Also, placental EV uptake seems to be an energy- and size-dependent process with decreased uptake rates at low temperatures and slower uptake of large EVs, which might affect EV-mediated signaling in placental pathophysiologies (Feng et al, 2022).

The EV–cell communication leads to phenotypic and functional changes in the recipient cell, which is determined by the EVs’ cargo. For example, non-coding RNAs such as miRNAs and long non-coding RNAs have been identified within placental EVs (Luo et al, 2009; Morales-Prieto et al, 2020). These may induce epigenetic modifications such as DNA methylation (Tedford et al, 2023).

To date, experimental approaches to study the functional role of EVs in biological setting are limited and include e.g., the inhibition of EV biogenesis (Catalano and O’Driscoll, 2020; Gurunathan et al, 2021). However, since this approach affects EV biogenesis in all tissues, it lacks specificity with regard to EVs released by specific tissue types such as the placenta and camouflages the interpretation of the results. Yet, a variety of functions have been attributed to placental EVs and it is believed that they play an integral role for healthy pregnancy progression (Salomon et al, 2013; Stenqvist et al, 2013; Tong et al, 2016), which is highlighted by the surge of placental EVs throughout pregnancy (Salomon et al, 2014; Sarker et al, 2014; Buca et al, 2020). These include the regulation of maternal homeostasis during implantation (Godakumara et al, 2021), the maintenance of pregnancy (Pap et al, 2008) and the onset of parturition (Menon et al, 2020). Noteworthy, limitations resulting from the different isolation methods and terminology of EVs during pregnancy have recently been unearthed (Barnes et al, 2023). These impair direct comparison and interpretation of insights on EVs during pregnancy between studies. We here summarized wide accepted key functions of placental EVs in the physiological modulations of pregnancy in Table 1. Similar to microchimeric cells, placental EVs bear great potential for tissue reparative and also regenerative effects (Fig. 3). In a mice model of multiple sclerosis, treatment with EVs from placenta-derived mesenchymal stem cells improved motor function and reduced DNA damage in oligodendroglia, along with an increased myelination within the spinal cord (Clark et al, 2019). In addition, placental-derived EVs also communicate with the maternal immune system. For example, the placenta harbors a unique profile of MHC molecules consisting of the classical polymorphic HLA-C and the non-classical oligomorphic HLA-E, -F, and -G molecules, which ensures pregnancy-maintaining crosstalk with the maternal immune system. Placental EVs also carry these HLA molecules and contribute to this crosstalk by interacting with the corresponding receptors on T cells (Pap et al, 2008), B cells (Song et al, 2023) and especially NK cells (Rebmann et al, 2016).

**Table 1:** Key functions of EVs associated with placental tissue in healthy pregnancies.

Function	Mechanism	Molecule	EV type	Source of EVs	Reference
**Immunomodulation**	Downregulation of NK cells, CD8 + T cells and decrease of γδ-T cell cytotoxicity (in vitro)	NKG2D ligand	sEVs	Placental explant (human)	(Hedlund et al, 2009)
	Downregulation of T-cell signaling (CD3-zeta and JAK3) and induction of apoptosis of leukocytes (in vitro)	FAS-L, PD-L1	sEVs	Placental EVs out of maternal blood (human)	(Sabapatha et al, 2006)
	Apoptosis of T cells and activated PBMCs (in vitro)	Fas-L, TRAIL	sEVs	Placental explant (human)	(Stenqvist et al, 2013)
	Downregulation of TNF-α production (in vitro)	miRNA-519c (C19MC member)	sEVs	Placental explant (human)	(Tiozzo et al, 2021)
	T_reg_ expansion and differentiation (in vitro)	HSPE1	mEVs	Trophoblastic cell line (human)	(Kovács Á et al, 2019)
	Activation of PBMC and promotion of cytokine production (in vitro)	Syncytin-1	lEVs	Placental explants (human)	(Holder et al, 2012)
	Promotion of release of proinflammatory cytokines from endothelial cells (in vitro)	Not investigated	sEVs	Plasma (human)	(Salomon et al, 2016)
	Induction of cytokine production (e.g., IL-8, CXCL1) in decidualized endometrial stromal fibroblasts (in vitro)	TNF-α associated activation of NF-κB signaling	sEVs	Placental explant (human)	(Taylor et al, 2020)
**Modulation of angiogenesis**	Promotion of extravillous trophoblast invasion (in vitro)	Oxygen tension	sEVs	Placental explant (human)	(Salomon et al, 2013)
	Vasodilatation of the endothelium (in vitro)	eNOS	sEVs, lEVs	Ex vivo placental perfusion (human)	(Motta-Mejia et al, 2017)
	Anti-constrictive and vasodilatory effects on blood vessels after 24 h (in vivo)	Not investigated	sEVs, lEVs	Pregnant mice	(Cheung et al, 2022)
	Decreased responsiveness to vasoconstrictor Angiotensin II (in vivo)	LOX-1-associated reduced AT1 expression	sEVs, lEVs	Ex vivo placental perfusion (human)	(Spaans et al, 2020)
**Modulation of metabolism**	Increased cholesterol synthesis in the liver (in vitro)	apoE	sEVs, mEVs, lEVs	Ex vivo placental perfusion (human)	(Tersigni et al, 2022)
	Improving maternal glucose tolerance (in vivo)	placental O-glycosyl transferase	sEVs	Maternal plasma (mice)	(Zierden et al, 2023)
	Glucose uptake and insulin sensitivity (in vitro)	miRNA	sEVs	Maternal plasma (human)	(Nair et al, 2021)
	Increase of insulin fasting concentration and increase of glucose-stimulated islet insulin secretion (in vivo)	Not investigated	sEVs	Maternal plasma (human)	(James-Allan et al, 2020)

*sEVs* small extracellular vesicles, *mEVs* middle-sized extracellular vesicles, *lEVs* large extracellular vesicles.

A number of studies have used placental tissue explants and trophoblast cell lines, which advance the understanding of the role of placental EVs during pregnancy. Due to the significant technical improvements allowing to isolate EVs, pivotal insights on placental EVs obtained from peripheral blood are emerging (Salomon et al, 2014; Brennan et al, 2020; Menon et al, 2020). This can be anticipated to excel our understanding of the physiological function of placental EVs in the near future. In addition, future advances in EV research will likely provide new avenues for functional EV studies, e.g., the inhibition of tissue-specific EV biogenesis. In this context the target tissue should likewise be taken into consideration, investigating to which degree one and the same (placental) EV induces functional changes in target cells of different tissues.

#### Fetal-derived EVs—beyond placental origin

Although the majority of pregnancy-associated EVs in the mother derive from placental cells, there is evidence to support that EVs can also originate from other fetal tissues, as proven by the transplacental transfer of intra-amniotically injected EVs in mice, which were previously isolated from human amnion epithelial cells (Sheller-Miller et al, 2016). Human amniotic fluid-derived EVs origin from a range of different cell types, such as amniotic fluid stem cells, amniotic mesenchymal stem cells, embryonic stem cells and placental cells (Gebara et al, 2022). In this context, health advantages have been attributed to mesenchymal stem and stromal cell-derived EVs due to their immunomodulatory, angiogenic, and regenerative properties (Giebel et al, 2017). For example, amniotic fluid-derived EVs may contribute to the amelioration of autoimmune diseases during pregnancy, as shown in the context of rheumatoid arthritis (Fig. 4) (Guthrie et al, 2010) or multiple sclerosis (Ponsonby et al, 2012). Recently, mesenchymal stem cells have been scrutinized as therapeutic mediators of pregnancy-related diseases, e.g., recurrent pregnancy loss or miscarriage (Li et al, 2021), suggesting that amniotic fluid-derived EVs may have similar effects. Noteworthy, fetal EVs—except placental EVs—originate most likely from cells that express classical HLA molecules, which may trigger a T-cell-dependent maternal alloimmune response (Buzas, 2023). We will address this issue later in the section on MMc-derived EVs.

### EV-mediated communication from mother to fetus

#### Transplacental transfer of maternal EVs

Similar to fetal EVs in the mother, very little is known about the functional role of maternal EVs in the fetus. Evidence for the ability of maternal EVs to pass the placental barrier was first provided through the mating of female mice with transgenic, tdTomato/green fluorescent male mice so that fetal tissues were detectable via tdTomato fluorescence (Sheller-Miller et al, 2019). In order to prove the passage of maternal EVs to the fetus, EVs were isolated from a human embryonic kidney cell line which was transfected with Cre recombinase. Subsequently, the EVs were intraperitoneally injected and could indeed be traced in the fetus, which was evident through the transition of the red to green fluorescence, which is only possible in the presence of Cre (Sheller-Miller et al, 2019). Besides that, it was recently shown for the first time in humans that also maternal microbiotal EVs of fecal origin could cross the placental barrier in mice and accumulate in the fetus (Kaisanlahti et al, 2023). Interestingly, the bacterial-derived EVs present in the amniotic fluid of healthy pregnant women share similar protein profiles and bacterial composition as bacterial EVs from maternal feces (Kaisanlahti et al, 2023). Additional insights on maternal EVs transmitted via the placental route to the fetus are currently not available, likely due to the lack of adequate biomarker or techniques to discriminate maternal from fetal EVs. However, there is intriguing first evidence of MMc-derived EVs in the fetus, as discussed below.

#### The echo of MMc: MMc-derived EVs

**Figure 6:**
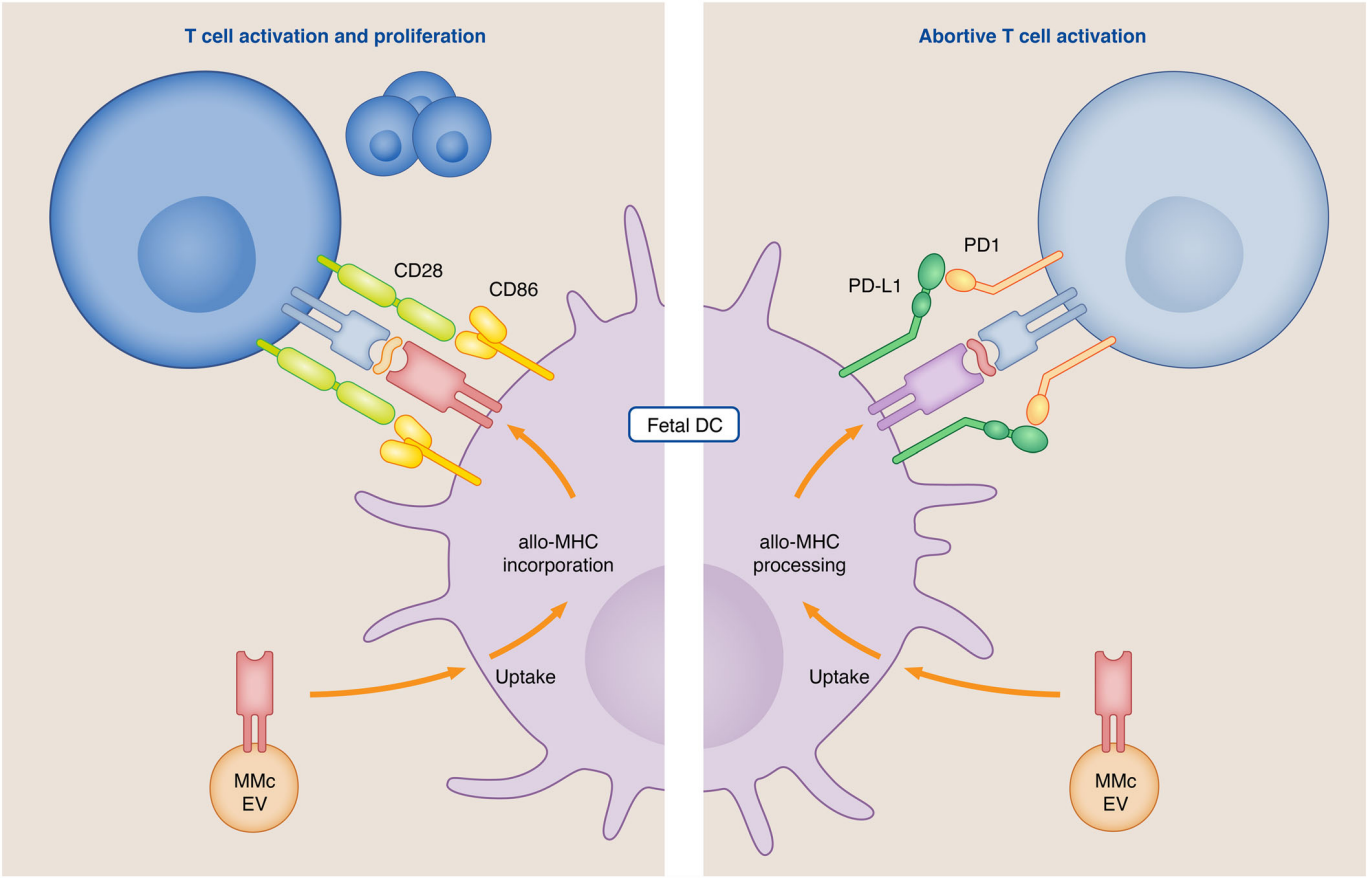
Induction of split tolerance by the presentation of MMc-derived EV. EVs released by MMc carry MHC molecules (red receptor), which can be taken up by antigen-presenting cells, where they are either incorporated into the plasma membrane (left) or processed and presented as antigen (right). The process of MHC molecule incorporation is referred to as cross-dressing and simultaneously leads to an upregulation and co-localization of CD86—an activator of proliferation (Bracamonte-Baran et al, 2017). This results in an activation of T cells upon T-cell receptor-specific (blue receptor) presentation of peptides (orange). Processing and presentation of the antigen (right) was associated with an emergence and co-localization of PD-L1—an inhibitor of cell proliferation (Bracamonte-Baran et al, 2017). As a result, interaction with T cells and their respective T-cell receptors (blue receptor) specific for these allopeptides (red peptide) led to an abortive activation. The selective upregulation and localization of co-stimulatory molecules depending on the MHC being cross-dressed or presented as allopeptide, leads to a divergent “split immune reaction”.

Interestingly, immune cells are capable of releasing EVs, hereby mediating immune functions. Outside the context of pregnancy, immune cell-derived EVs express allo- and immunogenic MHC type I and II molecules and can therefore be involved in the presentation of antigens (Raposo et al, 1996; Bracamonte-Baran et al, 2017; Buzas, 2023). As discussed above, a large fraction of MMc could be identified as immune cells (Loubière et al, 2006; Cuddapah Sunku et al, 2010; Stelzer et al, 2021) and indeed, the release of MMc-derived EVs, also carrying MHC molecules, could be confirmed. One pioneering study provided evidence for a role of MMc-derived EVs in antigen presentation upon uptake by antigen-presenting cells (APC) (Bracamonte-Baran et al, 2017). Here, proposed pathways of antigen presentation include “cross-dressing”—defined as the incorporation of the intact EV-derived MHC molecule into the membrane of an APC. Alternatively, EVs can be processed and presented as allopeptides (Bracamonte-Baran et al, 2017). This study associated the concept of immunological “split tolerance” as an outcome of the crosstalk between MMc-derived EVs and fetal immune cells (Fig. 6). The term split tolerance was first coined by Lustgraaf, describing the phenomenon that after tolerance induction towards chimeric cells, immune tolerance is subsequently abolished when re-exposed to an allograft from the same donor (Billingham et al, 1953; Lustgraaf et al, 1960). Hence “split tolerance” refers to diverging immune responses towards the same antigen within one host (Sprent et al, 1995). It can be postulated that first exposure to MMc carrying the maternal MHC not inherited by the fetus leads to a priming of the immune system, which at the timepoint of antigen re-exposure through the NIMA-expressing graft may trigger diverging immune responses. These findings generated in murine models raise important questions. The EVs’ signature of co-stimulatory or inhibitory molecules seems to be decisive for the fetal immune response towards NIMA (Fig. 6). Another factor that should be taken into consideration in future research endeavors is the MMc cell type, as MMc-derived  EVs may exhibit similar functions as their parent cell, as also shown in the context of CAR T-cell-derived EVs (Fu et al, 2019).

Besides engaging with the fetal immune system by presenting antigens or being presented as antigen and impacting on peripheral tolerance, it is tempting to speculate that MMc-derived EVs are also involved in the establishment of central tolerance. During lymphocyte development in the fetal thymus, thymic epithelial-derived EVs are known to contribute to the presentation of tissue-restricted antigens, possibly facilitating the negative selection (Skogberg et al, 2015; Lundberg et al, 2016). Similarly, maternal chimeric EVs could enter the thymic tissue and interfere with the lymphocytes’ learning process of self and non-self.

Beyond the interaction with the fetal immune system, MMc-derived EV can be involved in the replacement of lacking molecules. In mice born with a knockout for IL-2Ra (CD25), a gene involved in cell proliferation and differentiation, the lack of CD25 was reversed, presumably through MMc-mediated signaling. This study provides proof for the transfer of CD25 via soluble factors, most likely through the release of EVs (Wong et al, 2023).

To our knowledge, the other way around—the release of EVs by FMc in the mother—and the interaction with maternal tissues has not yet been investigated. However, EVs are involved in the maintenance of regulatory T cells, which also arise during pregnancy and promote tolerance towards the semi-allogenic fetus (Kinder et al, 2015). It is known that after a successful first pregnancy, second pregnancies have decreased risk of complications and increased success rates (Thiele et al, 2019). FMc or FMc-derived EVs may contribute to the maintenance of immunological memory in the context of pregnancy, subsequently promoting future pregnancy success.

## Concluding remarks

Both, microchimeric cells as well as chimeric EV are unique in their way of communication between mother and fetus. A broad repertoire of messages can be conveyed by single cells or even particles of cells. Deciphering these messages will advance our understanding of their function within the feto-maternal communicatome. However, the knowledge generated over the past few decades mainly results from correlative studies. This can be explained by the challenges to overcome technical limitation of functional approaches and need to be resolved by research endeavors in the upcoming years (pending issues).

Clearly, the immune system acts as a central sender and recipient of messages, highlighting its relevance within the communicatome. Both MC and EVs are powerful coding systems due to their biomolecular complexity. In the context of pregnancy, microchimeric cells are particularly relevant due to their longevity.

Pending issues

i. Identify the mechanisms by which chimeric cells communicate within their host.
ii. Identify mechanisms on the transport of chimeric cells across the placenta.
iii. In-depth evaluation and determination of novel marker for placental EV.
iv. Discovery of a marker or specific isolation technique for chimeric EVs.
v. Evaluate the role of fetal chimeric (non-placental) as well as maternal chimeric EVs, especially with the immune system.

## Glossary

**Allogenic**: Originating from another individual within the same species.
**Amniotic fluid**: Liquid filling the amniotic sac surrounding the fetus with protective functions.
**Chimerism**: The presence of genetically distinct cells or DNA, e.g., packed in extracellular vesicles, originating from another individual.
**Extracellular vesicles**: Nano- to micrometer-sized membrane-surrounded vesicles containing bioactive molecules, which are continuously released as messenger by each cell type of our body into the extracellular space.
**Fetal tolerance**: Processes ensuring that the fetus, which is in parts genetically different from its mother due to the expression of paternal antigens, is tolerated, and not rejected by the maternal immune system.
**Feto-maternal communicatome**: The entirety of coding systems and messages transferring biological information from mother to fetus and vice versa modulating maternal and fetal health.
**HLA molecules**: Gene complex, analog to the major histocompatibility complex, which codes for antigen-presenting receptors and regulate the immune response.
**Microchiome**: Entirety of trans- and intergenerational pregnancy-induced microchimeric cells as well as chimeric extracellular vesicles within an individual.
**Non-inherited maternal antigens (NIMA)**: Protein products of genes that are expressed by the mother but not inherited to the offspring by Mendelian inheritance.
**Placenta**: Transient organ of embryonic origin, which connects mother and fetus and selectively regulates the transfer of oxygen, nutrients, hormones and immune mediators.
**Regulatory T cells (T**_**reg**_**)**: T-cell subset, capable of regulating complex immune responses and by that establishing e.g., fetal tolerance and pregnancy success.
**Split tolerance**: Diverging immune responses towards the same antigen within one individual.

## References

[CR1] Albrecht M, Pagenkemper M, Wiessner C, Spohn M, Lütgehetmann M, Jacobsen H, Gabriel G, Zazara DE, Haertel C, Hecher K (2022). Infant immunity against viral infections is advanced by the placenta-dependent vertical transfer of maternal antibodies. Vaccine.

[CR2] Amenta PS, Gay S, Vaheri A, Martinez-Hernandez A (1986). The extracellular matrix is an integrated unit: ultrastructural localization of collagen types I, III, IV, V, VI, fibronectin, and laminin in human term placenta. Coll Relat Res.

[CR3] Ariga H, Ohto H, Busch MP, Imamura S, Watson R, Reed W, Lee TH (2001). Kinetics of fetal cellular and cell-free DNA in the maternal circulation during and after pregnancy: implications for noninvasive prenatal diagnosis. Transfusion.

[CR4] Arutyunyan A, Roberts K, Troulé K, Wong FCK, Sheridan MA, Kats I, Garcia-Alonso L, Velten B, Hoo R, Ruiz-Morales ER (2023). Spatial multiomics map of trophoblast development in early pregnancy. Nature.

[CR5] Auber M, Svenningsen P (2022). An estimate of extracellular vesicle secretion rates of human blood cells. J Extracell Biol.

[CR6] Badiavas EV (2004). The potential of bone marrow cells to orchestrate homeostasis and healing in skin. Blood Cells Mol Dis.

[CR7] Barnes MVC, Pantazi P, Holder B (2023). Circulating extracellular vesicles in healthy and pathological pregnancies: a scoping review of methodology, rigour and results. J Extracell Vesicles.

[CR8] Basiri M, Pahlavanneshan S (2021). Evaluation of placental alkaline phosphatase expression as a potential target of solid tumors immunotherapy by using gene and protein expression repositories. Cell J.

[CR10] Bianchi DW (1999). Fetal cells in the maternal circulation: feasibility for prenatal diagnosis. Br J Haematol.

[CR11] Bianchi DW, Zickwolf GK, Weil GJ, Sylvester S, DeMaria MA (1996). Male fetal progenitor cells persist in maternal blood for as long as 27 years postpartum. Proc Natl Acad Sci USA.

[CR12] Billingham RE, Brent L, Medawar PB (1953). Actively acquired tolerance of foreign cells. Nature.

[CR13] Boddy AM, Fortunato A, Wilson Sayres M, Aktipis A (2015). Fetal microchimerism and maternal health: a review and evolutionary analysis of cooperation and conflict beyond the womb. Bioessays.

[CR14] Bracamonte-Baran W, Florentin J, Zhou Y, Jankowska-Gan E, Haynes WJ, Zhong W, Brennan TV, Dutta P, Claas FH, van Rood JJ (2017). Modification of host dendritic cells by microchimerism-derived extracellular vesicles generates split tolerance. Proc Natl Acad Sci USA.

[CR15] Brennan K, Martin K, FitzGerald SP, O’Sullivan J, Wu Y, Blanco A, Richardson C, Mc Gee MM (2020). A comparison of methods for the isolation and separation of extracellular vesicles from protein and lipid particles in human serum. Sci Rep.

[CR16] Broestl L, Rubin JB, Dahiya S (2018). Fetal microchimerism in human brain tumors. Brain Pathol.

[CR17] Bryan JN (2015). Fetal microchimerism in cancer protection and promotion: current understanding in dogs and the implications for human health. AAPS J.

[CR18] Buca D, Bologna G, D’Amico A, Cugini S, Musca F, Febbo M, D’Arcangelo D, Buca D, Simeone P, Liberati M (2020). Extracellular vesicles in feto-maternal crosstalk and pregnancy disorders. Int J Mol Sci.

[CR19] Buzas EI (2023). The roles of extracellular vesicles in the immune system. Nat Rev Immunol.

[CR20] Catalano M, O’Driscoll L (2020). Inhibiting extracellular vesicles formation and release: a review of EV inhibitors. J Extracell Vesicles.

[CR21] Cheung S, Barrett C, Chen Q, Groom K, Chamley L, Lau SY (2022). First trimester placental extracellular vesicles likely contribute to the vasodilation of maternal resistance arteries in normal pregnancy. Placenta.

[CR22] Chiu HF, Chen CC, Tsai SS, Ho SC, Yang CY (2012). Parity, age at first birth, and risk of death from brain cancer: a population-based cohort study in Taiwan. BMC Public Health.

[CR23] Choi DS, Kim DK, Kim YK, Gho YS (2015). Proteomics of extracellular vesicles: exosomes and ectosomes. Mass Spectrom Rev.

[CR24] Cirello V, Recalcati MP, Muzza M, Rossi S, Perrino M, Vicentini L, Beck-Peccoz P, Finelli P, Fugazzola L (2008). Fetal cell microchimerism in papillary thyroid cancer: a possible role in tumor damage and tissue repair. Cancer Res.

[CR25] Clark K, Zhang S, Barthe S, Kumar P, Pivetti C, Kreutzberg N, Reed C, Wang Y, Paxton Z, Farmer D (2019). Placental mesenchymal stem cell-derived extracellular vesicles promote myelin regeneration in an animal model of multiple sclerosis. Cells.

[CR26] Conforti F, Pala L, Pagan E, Bagnardi V, De Pas T, Queirolo P, Pennacchioli E, Catania C, Cocorocchio E, Ferrucci PF (2021). Sex-based dimorphism of anticancer immune response and molecular mechanisms of immune evasion. Clin Cancer Res.

[CR27] Crewe C, Joffin N, Rutkowski JM, Kim M, Zhang F, Towler DA, Gordillo R, Scherer PE (2018). An endothelial-to-adipocyte extracellular vesicle axis governed by metabolic state. Cell.

[CR28] Cronqvist T, Erlandsson L, Tannetta D, Hansson SR (2020). Placental syncytiotrophoblast extracellular vesicles enter primary endothelial cells through clathrin-mediated endocytosis. Placenta.

[CR29] Cuddapah Sunku C, Gadi V, de Laval de Lacoste B, Guthrie KA, Nelson JL (2010). Maternal and fetal microchimerism in granulocytes. Chimerism.

[CR9] de bellefon LM, Heiman P, Kanaan SB, Azzouz DF, Rak JM, Martin M, Roudier J, Roufosse F, Lambert NC (2010). Cells from a vanished twin as a source of microchimerism 40 years later in a male with a scleroderma-like condition. Chimerism.

[CR30] Debnath K, Las Heras K, Rivera A, Lenzini S, Shin J-W (2023). Extracellular vesicle–matrix interactions. Nat Rev Mater.

[CR31] Dixon CL, Urrabaz-Garza R, Trivedi J, Menon R (2018). 606: placental alkaline phosphatase: Is it placenta-specific?. Am J Obstet Gynecol.

[CR32] Dubernard G, Aractingi S, Oster M, Rouzier R, Mathieu MC, Uzan S, Khosrotehrani K (2008). Breast cancer stroma frequently recruits fetal derived cells during pregnancy. Breast Cancer Res.

[CR33] Feng Y, Chen Q, Lau SY, Tsai BW, Groom K, Barrett CJ, Chamley LW (2022). The blocking of integrin-mediated interactions with maternal endothelial cells reversed the endothelial cell dysfunction induced by EVs, derived from preeclamptic placentae. Int J Mol Sci.

[CR34] Fu W, Lei C, Liu S, Cui Y, Wang C, Qian K, Li T, Shen Y, Fan X, Lin F (2019). CAR exosomes derived from effector CAR-T cells have potent antitumour effects and low toxicity. Nat Commun.

[CR35] Fujimoto K, Nakajima A, Hori S, Tanaka Y, Shirasaki Y, Uemura S, Irie N (2022). Whole-embryonic identification of maternal microchimeric cell types in mouse using single-cell RNA sequencing. Sci Rep.

[CR36] Gebara N, Scheel J, Skovronova R, Grange C, Marozio L, Gupta S, Giorgione V, Caicci F, Benedetto C, Khalil A (2022). Single extracellular vesicle analysis in human amniotic fluid shows evidence of phenotype alterations in preeclampsia. J Extracell Vesicles.

[CR37] Giebel B, Kordelas L, Börger V (2017). Clinical potential of mesenchymal stem/stromal cell-derived extracellular vesicles. Stem Cell Investig.

[CR38] Gilmore GL, Haq B, Shadduck RK, Jasthy SL, Lister J (2008). Fetal-maternal microchimerism in normal parous females and parous female cancer patients. Exp Hematol.

[CR39] Godakumara K, Ord J, Lättekivi F, Dissanayake K, Viil J, Boggavarapu NR, Faridani OR, Jääger K, Velthut-Meikas A, Jaakma Ü (2021). Trophoblast derived extracellular vesicles specifically alter the transcriptome of endometrial cells and may constitute a critical component of embryo-maternal communication. Reprod Biol Endocrinol.

[CR40] Gurunathan S, Kang MH, Qasim M, Khan K, Kim JH (2021). Biogenesis, membrane trafficking, functions, and next generation nanotherapeutics medicine of extracellular vesicles. Int J Nanomedicine.

[CR41] Guthrie KA, Dugowson CE, Voigt LF, Koepsell TD, Nelson JL (2010). Does pregnancy provide vaccine-like protection against rheumatoid arthritis?. Arthritis Rheum.

[CR42] Hall JM, Lingenfelter P, Adams SL, Lasser D, Hansen JA, Bean MA (1995). Detection of maternal cells in human umbilical cord blood using fluorescence in situ hybridization. Blood.

[CR43] Han C, Wang C, Chen Y, Wang J, Xu X, Hilton T, Cai W, Zhao Z, Wu Y, Li K (2020). Placenta-derived extracellular vesicles induce preeclampsia in mouse models. Haematologica.

[CR44] Harpavat S, Finegold MJ, Karpen SJ (2011). Patients with biliary atresia have elevated direct/conjugated bilirubin levels shortly after birth. Pediatrics.

[CR45] Harrington WE, Kanaan SB, Muehlenbachs A, Morrison R, Stevenson P, Fried M, Duffy PE, Nelson JL (2017). Maternal microchimerism predicts increased infection but decreased disease due to *Plasmodium falciparum* during early childhood. J Infect Dis.

[CR46] Haupt S, Caramia F, Klein SL, Rubin JB, Haupt Y (2021). Sex disparities matter in cancer development and therapy. Nat Rev Cancer.

[CR47] Hay FC, Hull MG, Torrigiani G (1971). The transfer of human IgG subclasses from mother to foetus. Clin Exp Immunol.

[CR48] Hedlund M, Stenqvist AC, Nagaeva O, Kjellberg L, Wulff M, Baranov V, Mincheva-Nilsson L (2009). Human placenta expresses and secretes NKG2D ligands via exosomes that down-modulate the cognate receptor expression: evidence for immunosuppressive function. J Immunol.

[CR49] Heidarzadeh M, Zarebkohan A, Rahbarghazi R, Sokullu E (2023). Protein corona and exosomes: new challenges and prospects. Cell Commun Signal.

[CR50] Hemberger M, Hanna CW, Dean W (2020). Mechanisms of early placental development in mouse and humans. Nat Rev Genet.

[CR51] Holder BS, Tower CL, Forbes K, Mulla MJ, Aplin JD, Abrahams VM (2012). Immune cell activation by trophoblast-derived microvesicles is mediated by syncytin 1. Immunology.

[CR52] Hoshino A, Costa-Silva B, Shen TL, Rodrigues G, Hashimoto A, Tesic Mark M, Molina H, Kohsaka S, Di Giannatale A, Ceder S (2015). Tumour exosome integrins determine organotropic metastasis. Nature.

[CR53] Huppertz B, Kaufmann P, Kingdom J (2002). Trophoblast turnover in health and disease. Fetal Maternal Med Rev.

[CR54] Iannotta D, Amruta A, Kijas AW, Rowan AE, Wolfram J (2024). Entry and exit of extracellular vesicles to and from the blood circulation. Nat Nanotechnol.

[CR55] James-Allan LB, Rosario FJ, Barner K, Lai A, Guanzon D, McIntyre HD, Lappas M, Powell TL, Salomon C, Jansson T (2020). Regulation of glucose homeostasis by small extracellular vesicles in normal pregnancy and in gestational diabetes. FASEB J.

[CR56] Johnson KL, Samura O, Nelson JL, McDonnell MDWM, Bianchi DW (2002). Significant fetal cell microchimerism in a nontransfused woman with hepatitis C: evidence of long-term survival and expansion. Hepatology.

[CR57] Jonsson AM, Uzunel M, Götherström C, Papadogiannakis N, Westgren M (2008). Maternal microchimerism in human fetal tissues. Am J Obstet Gynecol.

[CR58] Kaisanlahti A, Turunen J, Byts N, Samoylenko A, Bart G, Virtanen N, Tejesvi MV, Zhyvolozhnyi A, Sarfraz S, Kumpula S (2023). Maternal microbiota communicates with the fetus through microbiota-derived extracellular vesicles. Microbiome.

[CR59] Kamper-Jørgensen M, Biggar RJ, Tjønneland A, Hjalgrim H, Kroman N, Rostgaard K, Stamper CL, Olsen A, Andersen AM, Gadi VK (2012). Opposite effects of microchimerism on breast and colon cancer. Eur J Cancer.

[CR60] Kang M, Blenkiron C, Chamley LW (2023). The biodistribution of placental and fetal extracellular vesicles during pregnancy following placentation. Clin Sci.

[CR61] Kara RJ, Bolli P, Karakikes I, Matsunaga I, Tripodi J, Tanweer O, Altman P, Shachter NS, Nakano A, Najfeld V (2012). Fetal cells traffic to injured maternal myocardium and undergo cardiac differentiation. Circ Res.

[CR62] Kinder JM, Jiang TT, Ertelt JM, Xin L, Strong BS, Shaaban AF, Way SS (2015). Cross-generational reproductive fitness enforced by microchimeric maternal cells. Cell.

[CR63] Kinder JM, Stelzer IA, Arck PC, Way SS (2017). Immunological implications of pregnancy-induced microchimerism. Nat Rev Immunol.

[CR64] Knight M, Redman CW, Linton EA, Sargent IL (1998). Shedding of syncytiotrophoblast microvilli into the maternal circulation in pre-eclamptic pregnancies. Br J Obstet Gynaecol.

[CR65] Kovács ÁF, Fekete N, Turiák L, Ács A, Kőhidai L, Buzás EI, Pállinger É (2019). Unravelling the role of trophoblastic-derived extracellular vesicles in regulatory T cell differentiation. Int J Mol Sci.

[CR66] Kshirsagar SK, Alam SM, Jasti S, Hodes H, Nauser T, Gilliam M, Billstrand C, Hunt JS, Petroff MG (2012). Immunomodulatory molecules are released from the first trimester and term placenta via exosomes. Placenta.

[CR67] Kulaj K, Harger A, Bauer M, Caliskan ÖS, Gupta TK, Chiang DM, Milbank E, Reber J, Karlas A, Kotzbeck P (2023). Adipocyte-derived extracellular vesicles increase insulin secretion through transport of insulinotropic protein cargo. Nat Commun.

[CR68] Kupper N, Huppertz B (2022). The endogenous exposome of the pregnant mother: placental extracellular vesicles and their effect on the maternal system. Mol Aspects Med.

[CR69] Lambert N, Lee Nelson J (2003). Microchimerism in autoimmune disease: more questions than answers?. Autoimmun Rev.

[CR70] Lee GM, Johnstone B, Jacobson K, Caterson B (1993). The dynamic structure of the pericellular matrix on living cells. J Cell Biol.

[CR71] Leitner K, Szlauer R, Ellinger I, Ellinger A, Zimmer KP, Fuchs R (2001). Placental alkaline phosphatase expression at the apical and basal plasma membrane in term villous trophoblasts. J Histochem Cytochem.

[CR72] Leveque L, Hodgson S, Peyton S, Koyama M, MacDonald KP, Khosrotehrani K (2014). Selective organ specific inflammation in offspring harbouring microchimerism from strongly alloreactive mothers. J Autoimmun.

[CR73] Li H, Pinilla-Macua I, Ouyang Y, Sadovsky E, Kajiwara K, Sorkin A, Sadovsky Y (2020). Internalization of trophoblastic small extracellular vesicles and detection of their miRNA cargo in P-bodies. J Extracell Vesicles.

[CR74] Li Y-H, Zhang D, Du M-R (2021). Advances and challenges of mesenchymal stem cells for pregnancy-related diseases. Cell Mol Immunol.

[CR75] Li Z, Tao M, Huang M, Pan W, Huang Q, Wang P, Zhang Y, Situ B, Zheng L (2023). Quantification of placental extracellular vesicles in different pregnancy status via single particle analysis method. Clin Chim Acta.

[CR76] Lindtke D, Seefried FR, Drögemüller C, Neuditschko M (2023). Increased heterozygosity in low-pass sequencing data allows identification of blood chimeras in cattle. Anim Genet.

[CR77] Lishner M (2003). Cancer in pregnancy. Annal Oncol.

[CR78] Lo YM, Tein MS, Lau TK, Haines CJ, Leung TN, Poon PM, Wainscoat JS, Johnson PJ, Chang AM, Hjelm NM (1998). Quantitative analysis of fetal DNA in maternal plasma and serum: implications for noninvasive prenatal diagnosis. Am J Hum Genet.

[CR79] Loubière LS, Lambert NC, Flinn LJ, Erickson TD, Yan Z, Guthrie KA, Vickers KT, Nelson JL (2006). Maternal microchimerism in healthy adults in lymphocytes, monocyte/macrophages and NK cells. Lab Investig.

[CR80] Lundberg V, Berglund M, Skogberg G, Lindgren S, Lundqvist C, Gudmundsdottir J, Thörn K, Telemo E, Ekwall O (2016). Thymic exosomes promote the final maturation of thymocytes. Sci Rep.

[CR81] Luo S-S, Ishibashi O, Ishikawa G, Ishikawa T, Katayama A, Mishima T, Takizawa T, Shigihara T, Goto T, Izumi A (2009). Human villous trophoblasts express and secrete placenta-specific microRNAs into maternal circulation via exosomes1. Biol Reprod.

[CR82] Lustgraaf EC, Fuson RB, Eichwald EJ (1960). Sex tolerance and split tolerance. Plast Reconstr Surg.

[CR83] Madani G, Heiner DC (1989). Antibody transmission from mother to fetus. Curr Opin Immunol.

[CR84] Maloney S, Smith A, Furst DE, Myerson D, Rupert K, Evans PC, Nelson JL (1999). Microchimerism of maternal origin persists into adult life. J Clin Investig.

[CR85] Mathe G, Amiel JL, Schwarzenberg L, Cattan A, Schneider M (1963). Haematopoietic chimera in man after allogenic (homologous) bone-marrow transplantation. (Control of the secondary syndrome. specific tolerance due to the chimerism). Br Med J.

[CR86] Mathieu M, Martin-Jaular L, Lavieu G, Théry C (2019). Specificities of secretion and uptake of exosomes and other extracellular vesicles for cell-to-cell communication. Nat Cell Biol.

[CR87] Menon R, Debnath C, Lai A, Guanzon D, Bhatnagar S, Kshetrapal P, Sheller-Miller S, Salomon C (2020). Protein Profile changes in circulating placental extracellular vesicles in term and preterm births: a longitudinal study. Endocrinology.

[CR88] Mold JE, Michaëlsson J, Burt TD, Muench MO, Beckerman KP, Busch MP, Lee TH, Nixon DF, McCune JM (2008). Maternal alloantigens promote the development of tolerogenic fetal regulatory T cells in utero. Science.

[CR89] Morales-Prieto DM, Favaro RR, Markert UR (2020). Placental miRNAs in feto-maternal communication mediated by extracellular vesicles. Placenta.

[CR90] Motta-Mejia C, Kandzija N, Zhang W, Mhlomi V, Cerdeira AS, Burdujan A, Tannetta D, Dragovic R, Sargent IL, Redman CW (2017). Placental vesicles carry active endothelial nitric oxide synthase and their activity is reduced in preeclampsia. Hypertension.

[CR91] Muraji T, Masuya R, Harumatsu T, Kawano T, Muto M, Ieiri S (2022). New insights in understanding biliary atresia from the perspectives on maternal microchimerism. Front Pediatr.

[CR92] Nair S, Guanzon D, Jayabalan N, Lai A, Scholz-Romero K, Kalita de Croft P, Ormazabal V, Palma C, Diaz E, McCarthy EA (2021). Extracellular vesicle-associated miRNAs are an adaptive response to gestational diabetes mellitus. J Transl Med.

[CR93] Nassar D, Droitcourt C, Mathieu-d’Argent E, Kim MJ, Khosrotehrani K, Aractingi S (2012). Fetal progenitor cells naturally transferred through pregnancy participate in inflammation and angiogenesis during wound healing. FASEB J.

[CR94] Nelson JL (2012). The otherness of self: microchimerism in health and disease. Trends Immunol.

[CR95] Nelson JL, Gillespie KM, Lambert NC, Stevens AM, Loubiere LS, Rutledge JC, Leisenring WM, Erickson TD, Yan Z, Mullarkey ME (2007). Maternal microchimerism in peripheral blood in type 1 diabetes and pancreatic islet beta cell microchimerism. Proc Natl Acad Sci USA.

[CR96] Nguyen SL, Ahn SH, Greenberg JW, Collaer BW, Agnew DW, Arora R, Petroff MG (2021). Integrins mediate placental extracellular vesicle trafficking to lung and liver in vivo. Sci Rep.

[CR97] Nguyen Huu S, Oster M, Avril M-F, Boitier F, Mortier L, Richard M-A, Kerob D, Maubec E, Souteyrand P, Moguelet P (2009). Fetal microchimeric cells participate in tumour angiogenesis in melanomas occurring during pregnancy. Am J Pathol.

[CR98] Nguyen Huu S, Oster M, Avril MF, Boitier F, Mortier L, Richard MA, Kerob D, Maubec E, Souteyrand P, Moguelet P (2009). Fetal microchimeric cells participate in tumour angiogenesis in melanomas occurring during pregnancy. Am J Pathol.

[CR99] Noone AMHN, Krapcho M, Miller D, Brest A, Yu M, Ruhl J, Tatalovich Z, Mariotto A, Lewis DR, Chen HS, Feuer EJ, Cronin KA (2018) SEER cancer statistics review, 1975-2012. National Cancer Institute, Bethesda, MD. https://seer.cancer.gov/csr/1975_2015/, based on November 2017 SEER data submission, posted to the SEER web site, April 2018.

[CR100] O’Donoghue K (2008). Fetal microchimerism and maternal health during and after pregnancy. Obstet Med.

[CR101] Osada H, Doi S, Fukushima T, Nakauchi H, Seki K, Sekiya S (2001). Detection of fetal HPCs in maternal circulation after delivery. Transfusion.

[CR102] Ouyang Y, Bayer A, Chu T, Tyurin VA, Kagan VE, Morelli AE, Coyne CB, Sadovsky Y (2016). Isolation of human trophoblastic extracellular vesicles and characterization of their cargo and antiviral activity. Placenta.

[CR103] Owen RD (1945). Immunogenetic consequences of vascular anastomoses between bovine twins. Science.

[CR104] Page EW (1957). Transfer of materials across the human placenta. Am J Obstet Gynecol.

[CR105] Pap E, Pállinger E, Falus A, Kiss AA, Kittel A, Kovács P, Buzás EI (2008). T lymphocytes are targets for platelet- and trophoblast-derived microvesicles during pregnancy. Placenta.

[CR106] Ponsonby AL, Lucas RM, van der Mei IA, Dear K, Valery PC, Pender MP, Taylor BV, Kilpatrick TJ, Coulthard A, Chapman C (2012). Offspring number, pregnancy, and risk of a first clinical demyelinating event: the AusImmune Study. Neurology.

[CR107] Raposo G, Nijman HW, Stoorvogel W, Liejendekker R, Harding CV, Melief CJ, Geuze HJ (1996). B lymphocytes secrete antigen-presenting vesicles. J Exp Med.

[CR108] Rebmann V, König L, Nardi Fda S, Wagner B, Manvailer LF, Horn PA (2016). The potential of HLA-G-bearing extracellular vesicles as a future element in HLA-G immune biology. Front Immunol.

[CR109] Redman CW, Sargent IL (2000). Placental debris, oxidative stress and pre-eclampsia. Placenta.

[CR110] Sabapatha A, Gercel-Taylor C, Taylor DD (2006). Specific isolation of placenta-derived exosomes from the circulation of pregnant women and their immunoregulatory consequences. Am J Reprod Immunol.

[CR111] Salomon C, Kobayashi M, Ashman K, Sobrevia L, Mitchell MD, Rice GE (2013). Hypoxia-induced changes in the bioactivity of cytotrophoblast-derived exosomes. PLoS ONE.

[CR112] Salomon C, Scholz-Romero K, Sarker S, Sweeney E, Kobayashi M, Correa P, Longo S, Duncombe G, Mitchell MD, Rice GE (2016). Gestational diabetes mellitus is associated with changes in the concentration and bioactivity of placenta-derived exosomes in maternal circulation across gestation. Diabetes.

[CR113] Salomon C, Torres MJ, Kobayashi M, Scholz-Romero K, Sobrevia L, Dobierzewska A, Illanes SE, Mitchell MD, Rice GE (2014). A gestational profile of placental exosomes in maternal plasma and their effects on endothelial cell migration. PLoS ONE.

[CR114] Sarker S, Scholz-Romero K, Perez A, Illanes SE, Mitchell MD, Rice GE, Salomon C (2014). Placenta-derived exosomes continuously increase in maternal circulation over the first trimester of pregnancy. J Transl Med.

[CR115] Schepanski S, Chini M, Sternemann V, Urbschat C, Thiele K, Sun T, Zhao Y, Poburski M, Woestemeier A, Thieme MT (2022). Pregnancy-induced maternal microchimerism shapes neurodevelopment and behavior in mice. Nat Commun.

[CR116] Schmorl C (1893) Pathologisch-anatomische Untersuchungen uber Puerperal-Eklampsie. Verlag FCW Vogel

[CR117] Sedov E, McCarthy J, Koren E, Fuchs Y (2022). Fetomaternal microchimerism in tissue repair and tumor development. Dev Cell.

[CR118] Shao TY, Kinder JM, Harper G, Pham G, Peng Y, Liu J, Gregory EJ, Sherman BE, Wu Y, Iten AE (2023). Reproductive outcomes after pregnancy-induced displacement of preexisting microchimeric cells. Science.

[CR119] Sheller-Miller S, Choi K, Choi C, Menon R (2019). Cyclic-recombinase-reporter mouse model to determine exosome communication and function during pregnancy. Am J Obstet Gynecol.

[CR120] Sheller-Miller S, Lei J, Saade G, Salomon C, Burd I, Menon R (2016). Feto-maternal trafficking of exosomes in murine pregnancy models. Front Pharmacol.

[CR121] Skogberg G, Lundberg V, Berglund M, Gudmundsdottir J, Telemo E, Lindgren S, Ekwall O (2015). Human thymic epithelial primary cells produce exosomes carrying tissue-restricted antigens. Immunol Cell Biol.

[CR122] Smith NC, Brush MG, Luckett S (1974). Preparation of human placental villous surface membrane. Nature.

[CR123] Soncin F, Khater M, To C, Pizzo D, Farah O, Wakeland A, Arul Nambi Rajan K, Nelson KK, Chang CW, Moretto-Zita M (2018). Comparative analysis of mouse and human placentae across gestation reveals species-specific regulators of placental development. Development.

[CR124] Song P, Anna B, E Scott G, Chamley LW (2023). The interaction of placental micro-EVs with immune cells in vivo and in vitro. Am J Reprod Immunol.

[CR125] Spaans F, Quon A, Kirschenman R, Morton JS, Sawamura T, Tannetta DS, Sargent IL, Davidge ST (2020). Role of lectin-like oxidized LDL receptor-1 and syncytiotrophoblast extracellular vesicles in the vascular reactivity of mouse uterine arteries during pregnancy. Sci Rep.

[CR126] Sprent J, Hurd M, Schaefer M, Heath W (1995). Split tolerance in spleen chimeras. J Immunol.

[CR127] Stelzer IA, Urbschat C, Schepanski S, Thiele K, Triviai I, Wieczorek A, Alawi M, Ohnezeit D, Kottlau J, Huang J (2021). Vertically transferred maternal immune cells promote neonatal immunity against early life infections. Nat Commun.

[CR128] Stenqvist A-C, Nagaeva O, Baranov V, Mincheva-Nilsson L (2013). Exosomes secreted by human placenta carry functional Fas ligand and TRAIL molecules and convey apoptosis in activated immune cells, suggesting exosome-mediated immune privilege of the fetus. J Immunol.

[CR129] Stevens AM, Hermes HM, Rutledge JC, Buyon JP, Nelson JL (2003). Myocardial-tissue-specific phenotype of maternal microchimerism in neonatal lupus congenital heart block. Lancet.

[CR130] Straub RH (2007). The complex role of estrogens in inflammation. Endocr Rev.

[CR131] Taylor SK, Houshdaran S, Robinson JF, Gormley MJ, Kwan EY, Kapidzic M, Schilling B, Giudice LC, Fisher SJ (2020). Cytotrophoblast extracellular vesicles enhance decidual cell secretion of immune modulators via TNFα. Development.

[CR132] Tedford E, Badya NB, Laing C, Asaoka N, Kaneko S, Filippi BM, McConkey GA (2023). Infection-induced extracellular vesicles evoke neuronal transcriptional and epigenetic changes. Sci Rep.

[CR133] Tersigni C, Furqan Bari M, Cai S, Zhang W, Kandzija N, Buchan A, Miranda F, Di Simone N, Redman CW, Bastie C (2022). Syncytiotrophoblast-derived extracellular vesicles carry apolipoprotein-E and affect lipid synthesis of liver cells in vitro. J Cell Mol Med.

[CR134] Théry C, Witwer KW, Aikawa E, Alcaraz MJ, Anderson JD, Andriantsitohaina R, Antoniou A, Arab T, Archer F, Atkin-Smith GK (2018). Minimal information for studies of extracellular vesicles 2018 (MISEV2018): a position statement of the International Society for Extracellular Vesicles and update of the MISEV2014 guidelines. J Extracell Vesicles.

[CR135] Thiele K, Ahrendt LS, Hecher K, Arck PC (2019). The mnemonic code of pregnancy: comparative analyses of pregnancy success and complication risk in first and second human pregnancies. J Reprod Immunol.

[CR136] Thomas MR, Williamson R, Craft I, Yazdani N, Rodeck CH (1994). Y chromosome sequence DNA amplified from peripheral blood of women in early pregnancy. Lancet.

[CR137] Tiozzo C, Bustoros M, Lin X, Manzano De Mejia C, Gurzenda E, Chavez M, Hanna I, Aguiari P, Perin L, Hanna N (2021). Placental extracellular vesicles-associated microRNA-519c mediates endotoxin adaptation in pregnancy. Am J Obstet Gynecol.

[CR138] Tong M, Abrahams VM, Chamley LW (2018) Immunological effects of placental extracellular vesicles. *I*mmunol Cell Biol 10.1111/imcb.12049 10.1111/imcb.12049 29604098

[CR139] Tong M, Chamley LW (2018). Isolation and characterization of extracellular vesicles from ex vivo cultured human placental explants. Methods Mol Biol.

[CR140] Tong M, Chen Q, James JL, Wise MR, Stone PR, Chamley LW (2017). In vivo targets of human placental micro-vesicles vary with exposure time and pregnancy. Reproduction.

[CR141] Tong M, Kleffmann T, Pradhan S, Johansson CL, DeSousa J, Stone PR, James JL, Chen Q, Chamley LW (2016). Proteomic characterization of macro-, micro- and nano-extracellular vesicles derived from the same first trimester placenta: relevance for feto-maternal communication. Hum Reprod.

[CR142] Tong M, Stanley JL, Chen Q, James JL, Stone PR, Chamley LW (2017). Placental nano-vesicles target to specific organs and modulate vascular tone in vivo. Hum Reprod.

[CR143] Tóth E, Turiák L, Visnovitz T, Cserép C, Mázló A, Sódar BW, Försönits AI, Petővári G, Sebestyén A, Komlósi Z (2021). Formation of a protein corona on the surface of extracellular vesicles in blood plasma. J Extracell Vesicles.

[CR144] Tricarico C, Clancy J, D’Souza-Schorey C (2017). Biology and biogenesis of shed microvesicles. Small GTPases.

[CR145] Tsai BW, Lau S, Paek SY, Wise M, Kando I, Stone P, Chen Q, Chamley LW (2022). Antiphospholipid antibodies do not cause retargeting of placental extracellular vesicles in the maternal body. Placenta.

[CR146] Tyagi AM, Srivastava K, Mansoori MN, Trivedi R, Chattopadhyay N, Singh D (2012). Estrogen deficiency induces the differentiation of IL-17 secreting Th17 cells: a new candidate in the pathogenesis of osteoporosis. PLoS ONE.

[CR147] Vanzyl B, Planas R, Ye Y, Foulis A, de Krijger RR, Vives-Pi M, Gillespie KM (2010). Why are levels of maternal microchimerism higher in type 1 diabetes pancreas?. Chimerism.

[CR148] Wong JH, Sterns EE, Kopald KH, Nizze JA, Morton DL (1989). Prognostic significance of pregnancy in stage I melanoma. Archiv Surg.

[CR149] Wong VA, Dinh KN, Chen G, Wrenshall LE (2023) IL-2RαKO mice exhibit maternal microchimerism and reveal nuclear localization of IL-2Rα in lymphoid and non-lymphoid cells. Preprint at bioRxiv https://www.biorxiv.org/content/10.1101/2023.11.03.565571v2.full.pdf 10.3389/fimmu.2024.1369818 PMC11133634 38812502

[CR150] Yüzen D, Urbschat C, Schepanski S, Thiele K, Arck PC, Mittrücker HW (2023). Pregnancy-induced transfer of pathogen-specific T cells from mother to fetus in mice. EMBO Rep.

[CR151] Zhang Y, Tang Y, Liu Y, Wang J, Shen Y, Sun X, Kang M, Zhao M, Chen Q (2022). The autocrine role of placental extracellular vesicles from missed miscarriage in causing senescence: possible pathogenesis of missed miscarriage. Cells.

[CR152] Zierden HC, Marx-Rattner R, Rock KD, Montgomery KR, Anastasiadis P, Folts L, Bale TL (2023). Extracellular vesicles are dynamic regulators of maternal glucose homeostasis during pregnancy. Sci Rep.

